# Is Oxidative Stress the Link Between Cerebral Small Vessel Disease, Sleep Disruption, and Oligodendrocyte Dysfunction in the Onset of Alzheimer’s Disease?

**DOI:** 10.3389/fphys.2021.708061

**Published:** 2021-08-25

**Authors:** Ana Lloret, Daniel Esteve, Maria Angeles Lloret, Paloma Monllor, Begoña López, José Luis León, Ana Cervera-Ferri

**Affiliations:** ^1^INCLIVA, CIBERFES, Department of Physiology, Faculty of Medicine, University of Valencia, Valencia, Spain; ^2^Department of Clinical Neurophysiology, Hospital Clínico Universitario de Valencia, Valencia, Spain; ^3^Department of Neurology, Hospital Clínico Universitario de Valencia, Valencia, Spain; ^4^Departament of Neuroradiology, Ascires Biomedical Group, Hospital Clinico Universitario, Valencia, Spain; ^5^Department of Anatomy and Human Embryology, University of Valencia, Valencia, Spain

**Keywords:** demyelination, sleep dysfunction, blood-brain barrier permeability, ApoE4 and AD risk, vessel dysfunction, oligodendrocyte precursor cell, reactive oxygen species, beta-amyloid

## Abstract

Oxidative stress is an early occurrence in the development of Alzheimer’s disease (AD) and one of its proposed etiologic hypotheses. There is sufficient experimental evidence supporting the theory that impaired antioxidant enzymatic activity and increased formation of reactive oxygen species (ROS) take place in this disease. However, the antioxidant treatments fail to stop its advancement. Its multifactorial condition and the diverse toxicological cascades that can be initiated by ROS could possibly explain this failure. Recently, it has been suggested that cerebral small vessel disease (CSVD) contributes to the onset of AD. Oxidative stress is a central hallmark of CSVD and is depicted as an early causative factor. Moreover, data from various epidemiological and clinicopathological studies have indicated a relationship between CSVD and AD where endothelial cells are a source of oxidative stress. These cells are also closely related to oligodendrocytes, which are, in particular, sensitive to oxidation and lead to myelination being compromised. The sleep/wake cycle is another important control in the proliferation, migration, and differentiation of oligodendrocytes, and sleep loss reduces myelin thickness. Moreover, sleep plays a crucial role in resistance against CSVD, and poor sleep quality increases the silent markers of this vascular disease. Sleep disruption is another early occurrence in AD and is related to an increase in oxidative stress. In this study, the relationship between CSVD, oligodendrocyte dysfunction, and sleep disorders is discussed while focusing on oxidative stress as a common occurrence and its possible role in the onset of AD.

## Introduction

Alzheimer’s disease (AD) is a multifactorial neurodegeneration with its etiology still remaining unknown. Its complexity and multiple factors, which are constantly proven to influence its development, hinder the discovery of a curative or preventive treatment. Therefore, reviewing the events that take place, especially at the onset of the disease, and raising new hypotheses regarding their links is immensely relevant. The present review is devoted to elucidating the possibility of a connection between early events related to the onset of AD, including oxidative stress, oligodendrocyte dysfunction, cerebrovascular small disease (CVSD), and sleep disorders.

## Oxidative Stress Is an Early Event in AD Physiopathology

Although free radicals are traditionally considered damaging the by-products of cellular metabolism, at present, it has become known that the production of reactive oxygen species (ROS) and reactive nitrogen species (RNS) are involved in the regulation of processes that are central to most cell signaling. During physiological and pathophysiological processes, ROS and RNS act as secondary messengers which control the expression of different genes and are involved in multiple cellular events such as Ca^2+^ and redox homeostasis, chemotaxis, cell growth, cell cycle, cell adhesion, and apoptosis, among others (Dröge, 2002; Poljsak and Milisav, 2012). Owing to these reasons, when the production of free radicals exceeds the antioxidant cellular capacity, oxidative stress occurs (Sies, 1986), thereby leading to the possible deregulation of several cellular mechanisms.

Alzheimer’s disease is not an exception. Free radicals are involved in many pathological cascades as both beta-amyloid peptide (Aβ) and p-tau increase ROS levels. Oxidative stress is a proven process in the early onset of AD (Nunomura et al., 2001).

However, antioxidant therapy fails to treat AD. There could be several reasons for this: on the one hand, the vulnerability of neurons to oxidative stress varies between areas, and on the other hand, ROS generation is cell-specific (Bonizzi et al., 2000) and the brain is formed by many different cell types with diverse functions. In terms of the difference in vulnerability, it has been shown that there are areas, such as hippocampal CA1 and amygdala, which are specifically affected during the early onset of AD, and these areas are vulnerable to oxidative damage. The pyramidal neurons in the CA1 region undergo massive cell death under oxidative stress conditions (Wilde et al., 1997; Vornov et al., 1998; Sarnowska, 2002; Wang et al., 2005; Bearden et al., 2009; Cruz-Sánchez et al., 2010; Chang et al., 2012; Huang et al., 2012, 2013; Uysal et al., 2012). In addition, the amygdala and prefrontal cortex are more vulnerable to oxidative effects (Masood et al., 2008; Salim et al., 2010a,b, 2011a,b; Wang and Michaelis, 2010; Patki et al., 2013a,b; Solanki et al., 2015) than other cortical areas. In particular, oxidative stress triggers amygdalar hyperactivity and dendritic shrinking (Wellman, 2001; Vyas et al., 2002; Kreibich and Blendy, 2004; Brown et al., 2005; Radley et al., 2006; Wood et al., 2010) and may further potentiate synaptic disturbances by disrupting the hippocampus–amygdala projections. Interestingly, this vulnerability of the hippocampal CA1 and amygdala to oxidative stress could offer an appealing explanation for the very early damage caused to these areas due to AD.

## Oxidative Stress and CVSD in AD

Cerebrovascular small disease is a disorder that attacks the small cerebral arteries and microvessels. Its pathological presentation includes white matter hyperintensities, cerebral microbleeds, small subcortical infarct, enlarged perivascular space, brain atrophy, and lacunes visible through magnetic resonance imaging (Wardlaw et al., 2013). Vascular dysfunction is integral to the AD etiology and pathophysiology, and it includes blood–brain barrier (BBB) impairment and hemodynamic dysfunction (Klohs, 2020; Parodi-Rullán et al., 2020). CSVD also has a predictive effect on AD risk among the elderly people, and there are several epidemiological, genetic, and clinical studies related to both pathologies (Kim et al., 2020; Saridin et al., 2020). However, whether CSVD is a cause or consequence of AD pathology still remains a controversial issue. Recently, the evidence of imaging techniques suggests that intra-brain vascular dysregulation is an early pathological event during the disease development (Iturria-Medina et al., 2016). White matter hyperintensities predict an accelerated cognitive decline, increase in total cerebrospinal fluid (CSF) tau, hippocampal atrophy, and increased risk for AD (Kuller et al., 2003; Hertze et al., 2013; Bilello et al., 2015; Fiford et al., 2017; Salvadó et al., 2019). Moreover, in preclinical AD, white matter hyperintensity is observed before Aβ uptake and predicts its increase (Grimmer et al., 2012; Kandel et al., 2016). Furthermore, apolipoprotein E4 (ApoE4) carriers (that have a noticeable genetic risk factor to develop AD) have increased cerebrovascular amyloid angiopathy (Yu et al., 2015) that is caused by Aβ deposition in the vessel walls.

At present, the etiology of CVSD is unclear despite oxidative stress being the most widely supported theory which could explain the vascular dysfunction related to AD. The source of these ROS may increase the Aβ levels formed in the vessel walls or cause the dysfunction of endothelial cells (ECs). Endothelium cells contain a high quantity of mitochondria that produce high ROS levels in CVSD. These show upregulated xanthine oxidase, lipoxygenase, myeloperoxidase, and NADPH oxidases (NOX) enzymes (Doughan et al., 2008; Nazarewicz et al., 2013; Dikalov et al., 2014; Schulz et al., 2014; Sahoo et al., 2016), which also increase ROS production (Cervantes Gracia et al., 2017). The increase in ROS could culminate with an increased vessel permeability, BBB disruption (Kuriakose et al., 2019; He et al., 2020), and blood flow alteration (Freeman and Keller, 2012). Interestingly, these pathological processes have been demonstrated in AD (Klohs, 2020; Parodi-Rullán et al., 2020). This could indicate oxidative stress as the possible link between the early onset of CVSD and AD.

In recent years, it has been revealed that BBB disruption is an early marker of cognitive impairment (Montagne et al., 2015; Van De Haar et al., 2016a,b; Sweeney et al., 2018, 2019; Nation et al., 2019). The permeability of BBB increases during the very early phases of AD, and it also displays a leakage and breakdown. Besides, ApoE4 carriers show degeneration of brain pericytes, essential cells for BBB integrity (Zipser et al., 2007; Armulik et al., 2010; Bell et al., 2012; Halliday et al., 2016; Nikolakopoulou et al., 2019), and an accelerated BBB breakdown in both the hippocampus and medial temporal lobe (Montagne et al., 2020). The BBB breakdown contributes to cognitive decline in ApoE4 carriers in a manner that is independent of Aβ and tau levels. Oxidative stress could be a strong candidate for understanding early BBB disruption through cyclophilin A–matrix metalloproteinase-9 (CypA–MMP9) pathway activation (Jin et al., 2000; Gu et al., 2011). In fact, CypA-MMP9 is activated in degenerating brain capillary pericytes in ApoE4 carriers as revealed in brain tissue analysis (Halliday et al., 2016).

## Oxidative Stress and Oligodendrocytes in AD

Oligodendrocytes are the myelin-forming cells in the CNS; myelin is the high lipid content sheath that surrounds and electrically isolates axons. Oligodendrocytes are derived from oligodendrocyte precursor cells (OPCs) that are produced in development and throughout adult life (Dawson et al., 2003; Richardson et al., 2011). Oligodendrocytes, especially OPCs, are highly vulnerable to oxidative damage compared with other brain cells as, after an oxidative insult, newly differentiated oligodendrocytes are damaged earlier than their counterparts (Giacci and Fitzgerald, 2018). This vulnerability could be due to a poor protective mechanism against oxidative stress in these cells. Oligodendrocytes present a lower level of antioxidant defense (Thorburne and Juurlink, 1996) and a reduced DNA repair capacity based on the non-homologous end-joining (NHEJ), which is error-prone (Spaas et al., 2021). Moreover, oxidative stress disrupts oligodendrocyte differentiation by persistent histone acetylation (French et al., 2009) and by affecting the expression of mitochondrial key genes, such as NRF2 and PPAR-γ (De Nuccio et al., 2020). Following this, under oxidative damage conditions, myelin renewal is compromised (Giacci and Fitzgerald, 2018) and may also contribute to the demyelinating phenotype observed during early AD (Sachdev et al., 2013). Demyelination due to oxidative damage has been broadly demonstrated in neurodegenerative disorders such as multiple sclerosis (Lassmann and van Horssen, 2016). However, experiments with Aβ peptide *in vitro* are controversial. It has been reported that Aβ causes demyelination (Horiuchi et al., 2012) in primary cultures incubated with 1 mM Aβ, and other studies report that Aβ causes remyelination with a similar concentration (Quintela-López et al., 2019). Nevertheless, in these studies, oxidative stress, which could be very important to enlighten these concepts, has not been measured.

Oligodendrocytes are also very sensitive to excitotoxicity given their high contents of AMPA and NMDA receptors which, by altering mitochondrial Ca^2+^ levels, makes them more vulnerable to oxidative stress than neurons (Ibarretxe et al., 2006). In the early phases of AD, both excitotoxicity and oxidative stress have been observed (Campbell and Tobler, 1984; Olney et al., 1997; Cutler et al., 2004), and their additive effect could be deleterious for oligodendrocytes.

## Oxidative Stress and Sleep Disruption in AD

Right from invertebrates to superior vertebrates, all animals sleep. This indicates that it is a conserved process in evolution (Cirelli and Tononi, 2008; Joiner, 2016). Humans sleep nearly one-third of their lives and, in this process, several special circumstances occur for both vessels and oligodendrocytes as well as for the expression of antioxidant enzymes (discussed later). Proper sleep is essential for crucial brain functions such as memory consolidation and detoxification of waste products (Lloret et al., 2020). Therefore, it is not surprising that lack of sleep can have detrimental effects on brain homeostasis. One of the consequences of sleep deprivation is an increase in brain oxidative stress, either through the increase in the generation of free radicals or through the decrease in antioxidant levels (Everson et al., 2005; Villafuerte et al., 2015), especially in the hippocampus. However, some studies do not show any increase in oxidative stress following sleep deprivation in animal models; however, they all undergo acute sleep deprivation (D’Almeida et al., 1997; Cirelli et al., 1999; Gopalakrishnan et al., 2004). Nevertheless, studies in chronic sleep restriction with *Drosophila* mutants suggest that one function of sleep is to increase the resistance of organism to oxidative stress (Hill et al., 2018). Other studies, also on *Drosophila*, have revealed that an increase in oxidative damage causes breakdown of sleep, which implies that both phenomena are linked in a bidirectional manner: oxidative stress causes fragmented sleep and fragmented sleep increases oxidative stress (Koh et al., 2006).

Oxidative stress and sleep disorders have also been related in humans. Obstructive sleep apnea syndrome causes oxidative stress coinciding with each episode of apnea, measured with continuous monitoring during sleep (Lloret et al., 2007). In postmenopausal women complaining of insomnia, lipid peroxidation is increased (Hachul De Campos et al., 2006). Moreover, it has been recently reported that night workers have higher levels of oxidative stress damage and lower levels of antioxidant defenses than the day workers (Teixeira et al., 2019).

Patients with Alzheimer’s disease show a high prevalence and severity of sleep disturbances that seem to appear several years prior to the onset of cognitive decline (Lloret et al., 2020). Furthermore, ApoE4 carriers undergo a 2-fold increase in the odds of sleep-disordered breathing (Kadotani et al., 2001). These also cause poor sleep quality (Drogos et al., 2016). In contrast, better sleep consolidation seems to attenuate incidence of AD linked to APOE ε4 impact, and there is no association with age compared with APOE ε4 non-carriers (Lim et al., 2013b; Pan et al., 2021). A multicenter study showed that midlife and late-life insomnia are associated with a higher risk of late-life dementia (Sindi et al., 2018). However, the detrimental effect of sleep deprivation could also be taking place over a short term as healthy people suffering from poor sleep quality present cognitive impairment only after 1 year (Potvin et al., 2012). Moreover, high sleep fragmentation increases the risk of developing AD by 1.5 times after 6 years (Lim et al., 2013a). Following this line, a 40-year follow-up study including 1,574 men reported that sleep disturbance increases the risk of developing AD by 51% (Benedict et al., 2015). Furthermore, it has been suggested that the treatment of obstructive sleep apnea is capable of reducing the risk of dementia (Dunietz et al., 2021). This idea is supported by a recent meta-analysis reported by Bubu et al. (2017). For the explanation provided here, we can conclude that early oxidative stress in AD could provoke sleep disturbance, as well as sleep disorders, which could consequently lead to an increment in oxidative damage.

## Sleep and Oligodendrocytes

Sleep is crucial in the synthesis and maintenance of the myelin due to an expression cycle of genes during the sleep/wake period (Cirelli et al., 2004; Thompson et al., 2010). Oligodendrocytes and their precursors show a different pattern of sleep-related expression of genes (Bellesi et al., 2013). Indeed, genes involved in phospholipid synthesis and myelination promoting OPC proliferation are preferentially transcribed during sleep, whereas genes implicated in apoptosis, cellular stress response (including many antioxidant enzymes), and OPC differentiation are enriched during wakefulness. Specifically, OPC proliferation doubles during sleep, correlating with the REM phase, whereas an OPC differentiation is higher during the waking state (Bellesi et al., 2013). Moreover, there is a reduction in myelin thickness following sleep loss with no changes in the internodal length (Bellesi et al., 2018). In conclusion, oligodendrocytes may express a particular vulnerability to sleep loss owing to their high susceptibility to incur oxidative stress. AD patients present high levels of sleep disruption very early, it is expected that oligodendrocytes are affected for this reason.

## Oligodendrocytes and CSVD

The function of oligodendrocytes depends on proper blood perfusion, as hypoperfusion interferes with white matter repair by disrupting OPC renewal mechanisms (Miyamoto et al., 2013). Accordingly, in mice expressing human ApoE4, a decrease in the white matter levels is accompanied by microvasculature injury (Koizumi et al., 2018). ECs physiologically secrete factors that promote OPC proliferation. Furthermore, in low-oxygen environments, ECs release various factors into extracellular vesicles to increase OPC survival (Kurachi et al., 2016). Other studies have identified that this relationship is bidirectional as OPC also supports ECs, releasing signaling factors involved in BBB maintenance from OPCs to ECs (Seo et al., 2014). In order to migrate, OPCs use vessels as scaffolding (Snapyan et al., 2009), interacting specifically with pericytes (Maki et al., 2015). In this context, a decrease in the number of pericytes in the brains of patients with AD has been demonstrated (Schultz et al., 2018). Therefore, both cells, endothelial and oligodendrocytes, are very sensitive to oxidative damage and are equally related to a crossing-pathway dysfunction in AD. Hence, oxidative stress is the chief participant in the relationship between oligodendrocyte dysfunction, subsequent demyelination, and CVSD due to its early occurrence in AD.

## Sleep and CVSD

Sleep and brain vascular functionality, specifically BBB function, are closely related. In fact, night sleep regulates substance transport all across and along the BBB. Sleep loss increases the BBB permeability, and sleep disruption can also lead to BBB breakdown (Hurtado-Alvarado et al., 2017).

It has been demonstrated that CSVD is associated with sleep disorders. Fifty-four percent of patients with CSVD suffer from chronic insomnia (Wang et al., 2019). Moderate-to-severe obstructive sleep apnea is also associated with CSVD (Song et al., 2017), and non-breathing-related sleep fragmentation is also common and related to the pathological markers in patients with CSVD (Wang J. et al., 2020).

**FIGURE 1:**
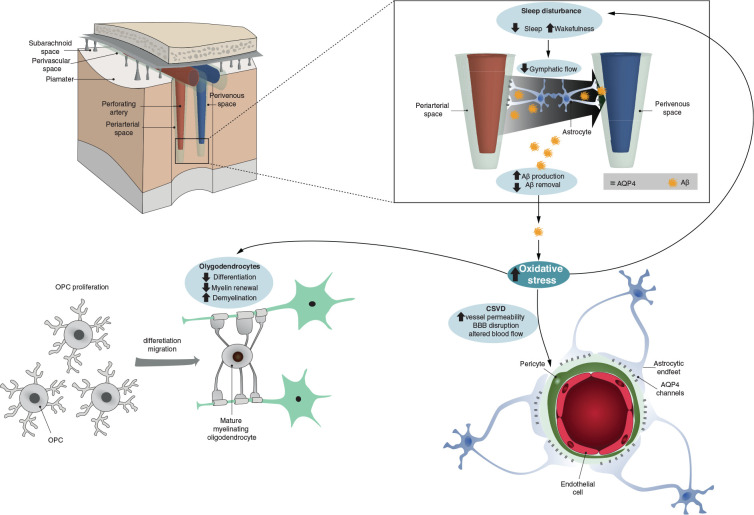
Schematic representation summarizing how oxidative stress could link vessel damage, sleep disruption, and oligodendrocyte dysfunction in early AD. The **top panels** represent brain perfusion with the glymphatic system detailed. The waste products including beta-amyloid are not properly cleared by the glymphatic system during sleep due to the disruption of latter caused by AD-related oxidative stress. Oxidative stress also increases CSVD-related alterations and oligodendrocyte/OPC unsuccessful differentiation (**bottom panel**).

One of the primary functions of sleep is to eliminate waste products from the brain. The term “glymphatic system” refers to the manner in which waste products coming from the interstitial fluid enter the brain parenchyma along with arterial perivascular spaces and exit through venous perivascular spaces (Iliff et al., 2012; Xie et al., 2013). Recent studies in mice have revealed that Aβ concentration in interstitial fluid fluctuates during the day and night (Kress et al., 2018). Its concentration peaks during the night and the loss of components of the molecular clock increases amyloid plaques formation. Hence, it is not surprising that sleep disturbance and CSVD could be important comorbidities for AD. In AD, both sleep disturbance and BBB dysfunction have been described, and both occur early on (Semyachkina-Glushkovskaya et al., 2020). Even healthy people carrying the ApoE4 allele show alterations in both processes. Therefore, sleep disruption and dysfunction with respect to the vascular functionality of the brain are two phenomena that are closely related and implicated in the onset of AD (see Figure 1 for a schematic summary).

## Is Oxidative Stress the Link Between Sleep, CVSD, and Oligodendrocytes Dysfunction in AD?

In this study, we hypothesized that oxidative stress could be the molecular link between oligodendrocyte and endothelium dysfunction and sleep disruption. The primary source of ROS in cells is mitochondria, and specifically, the electronic transport chain. In AD, several evidences of mitochondrial dysfunction and oxidative stress caused by Aβ peptides have been found: energy failure prior to the development of plaque pathology, ATP formation reduction, complexes I and IV inhibition that increases ROS production, and high levels of oxidized mtDNA (Cenini et al., 2019).

Reactive oxygen species are important physiological cell-signaling molecules in the vascular endothelium. Therefore, they are normally formed in these cells. However, this fact precisely makes them more vulnerable when they are formed in excess. Cerebral ECs also have a high concentration of mitochondria, which provides an increased opportunity for generating oxidative stress. It has been shown that BBB disruption could be caused by ROS-induced metalloproteinase (MMP) activity, which stimulates the degradation of tight junctions (Dawson et al., 2003). On the contrary, it has also been mentioned that increased NOXs activity may be involved in this process. NOXs transfer electrons from NADPH to oxygen, thereby generating superoxide radical. Moreover, it is involved in BBB breakdown in CVSD and AD (Freeman and Keller, 2012).

Oligodendrocytes and endothelium closely interact with each other. This oligovascular niche is important to sustain angiogenesis and oligodendrogenesis (Arai and Lo, 2009a,b). Oligodendrocytes are highly susceptible to oxidative stress, as they are the predominant iron-containing cells of the brain (Connor and Menzies, 1995) and have reduced levels of glutathione, glutathione peroxidase, and mitochondrial manganese superoxide dismutase (Thorburne and Juurlink, 1996). In ECs, ROS-activated MMPs induce oligodendrocyte dysfunction by degrading the extracellular protein matrix, consequently impairing their differentiation (Gorter and Baron, 2020). NOX enzymes also increase in oligodendrocytes through Aβ-induced NMDA receptors/PKC pathway (Cavaliere et al., 2013; Liu et al., 2019).

**FIGURE 2:**
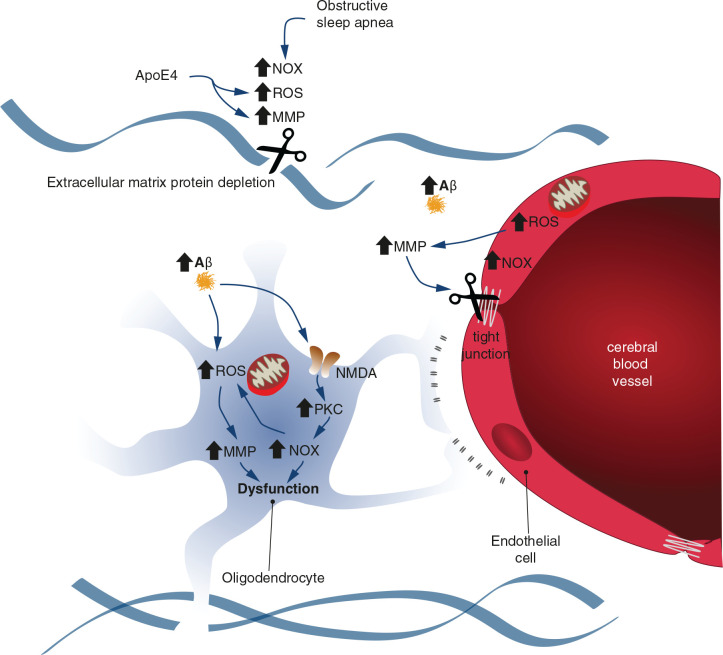
Schematic representation illustrating the common molecular pathways between endothelial and oligodendrocyte dysfunction and obstructive sleep apnea. Oxidative stress could increase the activity of metalloproteinases (MMPs) and NADPH oxidases (NOXs). The extracellular protein matrix and tight junctions are degraded by MMPs, which contributes to endothelial and oligodendrocyte dysfunction. The NOX activity provokes increased ROS levels in a positive feedback loop.

Finally, as mentioned previously, ROS are generated in obstructive sleep apnea due to intermittent hypoxic episodes resulting in sleep fragmentation (Nair et al., 2011a,b). Subsequently, ROS can directly activate MMPs and also *via* nuclear factor kappa B (NF-κB) pathway activation (Htoo et al., 2006). In fact, plasma MMP9 levels are elevated in patients with obstructive sleep apnea (Franczak et al., 2019a; Mashaqi et al., 2021), and urinary MMP2 activity has been shown to increase in accordance with obstructive sleep apnea severity (Franczak et al., 2019b). On the contrary, it has also been described that ROS induces the synthesis and stability of hypoxia-inducible factor 1α (HIF-1α) during hypoxic periods. In turn, HIF-1α increases the activity of NOXs, which are an important prooxidant, producing superoxide and H_2_O_2_ (Takac et al., 2011; Kaur et al., 2014; Wang N. et al., 2020). These changes promote a prooxidant state, disrupt hippocampal synaptic plasticity, and impair spatial memory in patients with obstructive sleep apnea (Arias-Cavieres et al., 2020). Considering the aforementioned changes, we concluded that oxidative stress could increase MMP and NOX activities that are involved in oligodendrocyte and endothelium dysfunction and sleep disruption. Figure 2 summarizes these common molecular pathways.

## Concluding Remarks

Considering the arguments so far, it is apparent that oligodendrocyte dysfunction, sleep, and CSVD are closely related to each other, and all constitute oxidative stress as a very early indicator. It is noteworthy that the major lesions in AD are Aβ and tau; thus, the onset of the disease should be related to the formation of these molecules in an exacerbated form. All the present models point out that the first positive biomarker in AD is soluble Aβ that probably begins its silent cell deregulation decades before any observable cognitive impairment (Lloret et al., 2019). Here, we proposed that Aβ could generate oxidative stress early on in AD, but only selectively affecting cells, especially vulnerable ones such as oligodendrocytes and ECs. Moreover, this oxidative stress would affect myelin formation and renewal, sleep, and vessel function (including BBB function). All these processes will interact with each other and show positive feedback.

## Author Contributions

AL has elaborated the items to be discussed and coordinated the work. AL and DE have written the first draft. AC-F has designed and drew the figures. JL and BL have found and selected the references. PM, AC-F, and ML have expanded and corrected the draft. All author listed have made a substantial, direct and intellectual contribution to the work, and approved it for publication.

## Conflict of Interest

The authors declare that the research was conducted in the absence of any commercial or financial relationships that could be construed as a potential conflict of interest.

## Publisher’s Note

All claims expressed in this article are solely those of the authors and do not necessarily represent those of their affiliated organizations, or those of the publisher, the editors and the reviewers. Any product that may be evaluated in this article, or claim that may be made by its manufacturer, is not guaranteed or endorsed by the publisher.
